# Quantum-Chemical Calculation and Visualization of the Vibrational Modes of Graphene in Different Points of the Brillouin Zone

**DOI:** 10.1186/s11671-015-0945-9

**Published:** 2015-07-11

**Authors:** Tetiana Lebedieva, Victor Gubanov, Galyna Dovbeshko, Denys Pidhirnyi

**Affiliations:** Department of Physics of Biological System, Institute of Physics, NAS of Ukraine, Prospect Nauky, 46, Kyiv, 03680 Ukraine; Physical Department, Taras Shevchenko National University of Kyiv, Volodymyrska Street, 64/13, Kyiv, 01601 Ukraine

**Keywords:** Quantum-chemical calculation, Brillouin zone, CARS, Discrete breather, Graphene, 33.15.Bh, 33.20.Tp, 63.22.Rc

## Abstract

Different notations of graphene irreducible representations and optical modes could be found in the literature. The goals of this paper are to identify the correspondence between available notations, to calculate the optical modes of graphene in different points of the Brillouin zone, and to compare them with experimental data obtained by Raman and coherent anti-Stokes Raman scattering (CARS) spectroscopy. The mechanism of the resonance enhancement of vibration modes of the molecules adsorbed on graphene in CARS experiments is proposed. The possibility of appearance of the discrete breathing modes is discussed.

## Background

Graphene consists of hexagonal rings of carbon atoms packed in periodic structure with symmetry D_6h_, and due to its electronic, mechanical, and other physical and chemical characteristics, it is of great interest for scientific community.

In spite of it, until now, there are different notations for symmetry of phonon modes presented in literature [1–3]. Here, we want to give two main used notations in literature [4, 5] for easy interpretation of Raman spectra and dispersion of graphene. Dispersion is not universally recognized characteristics in contrast to Raman spectra which is a passport for graphene and graphene-based materials. That is why we calculate, visualize, and compare the optical modes in graphene in different points of the Brillouin zone with Raman and coherent anti-Stokes Raman scattering (CARS) experiment data. We concluded about possible arising of new modes. Here, we discussed a resonant character of conventional Raman and CARS spectra for graphene.

## Methods

The technique of quantum operator of projection on projective representations, which describes elementary vibration modes, is used to determine the shapes of the normal modes analytically in different points of the Brillouin zone, including **k** ≠ 0. The shapes of the normal vibrational modes are computed in the *K*, *M*, and *Г* points of the Brillouin zone.

**Fig. 1:**
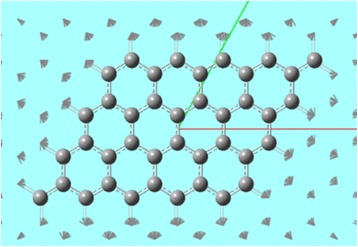
Modeled fragment of graphene monolayer

Optical modes of single-layer graphene were investigated using density functional theory (DFT) with periodic boundary conditions (PBC) and STO-3G basis with the correlation functional VWN5. As an initial atomic configuration fragment, a flat hexagonal lattice of 40 carbon atoms (Fig. 1) or 12 elementary hexagonal cells of graphene with symmetry type D_6h_ were modeled and optimized in the same basis and function. Parallelized implemented software at Gaussian 09 packages [6] was used for calculations. Program Gauss View 5.0 was used for visualization of vibrations.

## Results and Discussion

**Fig. 2:**
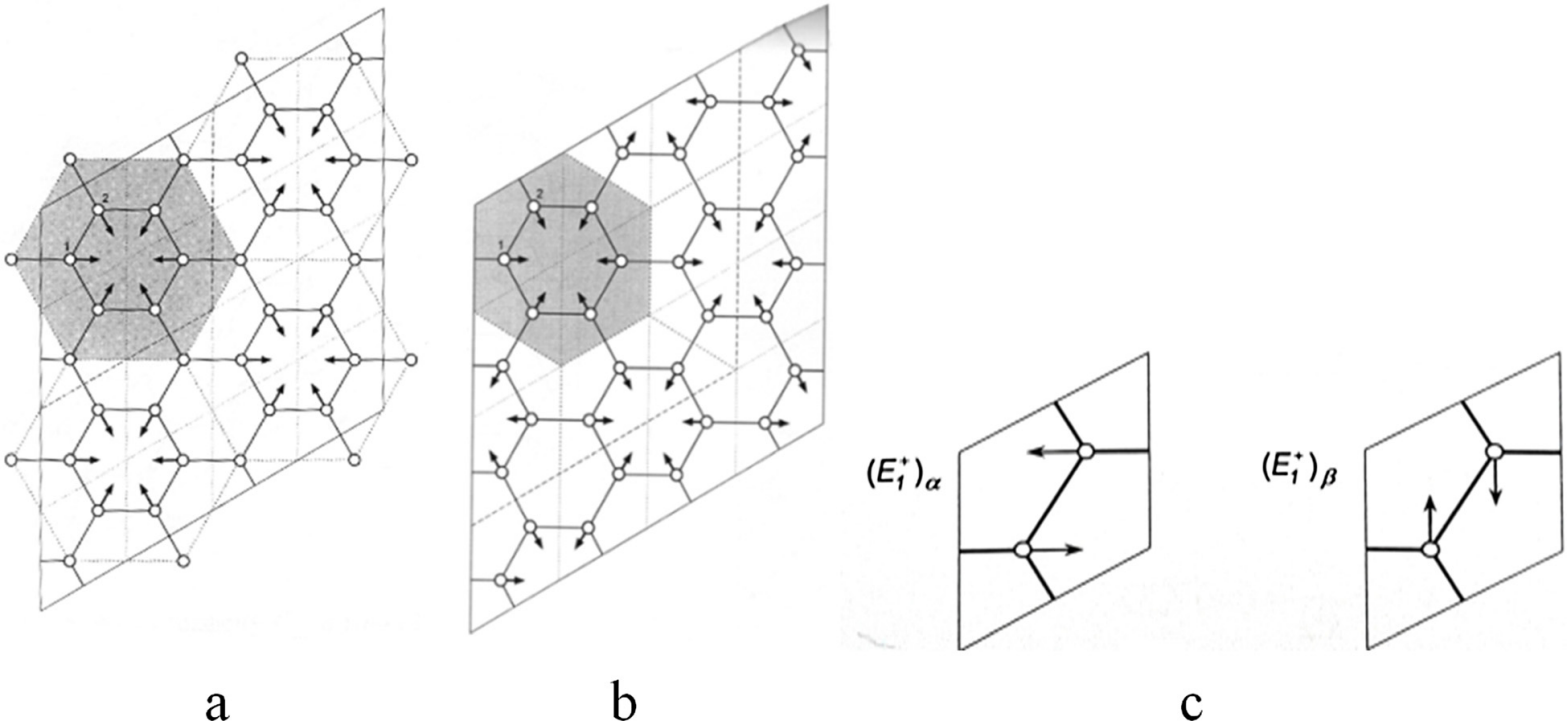
Forms of vibrations of a single-layer graphene at different points of the Brillouin zone (Table 1) [15]. **a** 1455. **b** 1500. **c** 1689 and 1705

Fundamental modes of graphene layer at the *Г* (D_6h_), *K* (D_3h_), and *M* (D_2h_) points of the Brillouin zone were identified by using the apparatus of the quantum-mechanical projection operator. To optimize the search of the characteristic frequencies, the discrepancies between the experimental and calculated frequencies of the vibrational spectra were analyzed. Analysis of carbon atom movements in graphene lattice for the main observed bands of the vibrational spectra was done (Fig. 2).

Phonon spectra of graphene in the Brillouin zone consist of six branches. Phonons with zero quasi-momentum at the *Г*-point (**k** = 0) or fundamental vibrational modes could be presented by irreducible representations of the D_6h_ group in the notations [2] by the following way:

$$ {\varGamma}_{\mathrm{vib}}={A}_2^{+}+{A}_3^{-}+{E}_1^{+}+{E}_2^{-} $$ [4], *Г*_vib_ = *B*_2g_ + *A*_2u_ + *E*_2g_ + *E*_1u_ [7] or $$ {\varGamma}_{\mathrm{vib}}={\varGamma}_2^{+}+{\varGamma}_3^{-}+{\varGamma}_5^{+}+{\varGamma}_{6\kern1.5em }^{-} $$ [5],

including the acoustic vibrations

$$ {\varGamma}_{\mathrm{ac}}={A}_3^{-}+{E}_2^{-},\ \Big({\varGamma}_{\mathrm{ac}}={A}_{2\mathrm{u}}+{E}_{1\mathrm{u}}\ \mathrm{or}\ {\varGamma}_{\mathrm{ac}}={\varGamma}_3^{-}+{\varGamma}_6^{-} $$

and optical ones

$$ {\varGamma}_{\mathrm{opt}}={A}_2^{+}+{E}_1^{+},\ {\varGamma}_{\mathrm{opt}}={B}_{2\mathrm{g}}+{E}_{2\mathrm{g}}\ \mathrm{or}\ {\varGamma}_{\mathrm{opt}}={\varGamma}_2^{+}+{\varGamma}_5^{+}. $$

Calculated vibrations at 1689 and 1705 cm^−1^ correspond to a double degenerated state with ${\left({E}_1^{+}\right)}_{\upalpha}$ and ${\left({E}_1^{+}\right)}_{\upbeta}$ according to the Bir and Pikus classification [2]. This mode could be experimentally observed near 1600 cm^−1^, so-called the G-mode at the *Г*-point of the Brillouin zone and named by Dresselhaus as E_2g_ mode. Calculated vibration at a frequency of 1455 cm^−1^ is associated with the *K*-point of the Brillouin zone and observed in the region of 1250–1380 cm^−1^ at different frequencies of laser excitation and called the D-mode (Table 1). The calculated vibration frequency at 1500 cm^−1^ is associated with the i-TO phonon at the *M*-point of the Brillouin zone and appeared in unideal graphene and on its boundaries and registered in CARS experiment at 1430 cm^−1^ [8].

**Table 1:** Experimental and calculated data of graphene monolayer

*ν*, cm^−1^ (calculated)	*ν*, cm^−1^ (experimental)	Point of the Brillouin zone	Mode
1455 (Fig. 2a)	1250–1380	*K* = *T*′ − *T*_1_′	D
1500 (Fig. 2b)	1430	*M*	i-TO phonon at the *M*-point
1689 (Fig. 2c)	1581	*Γ*	G
1705 (Fig. 2c)	1581	*Γ*	G

Let us describe elementary processes, which lead to appearance of different modes mentioned above. The most unusual vibration in graphite-like materials is so-called the 2D-mode, which was explained by Thomsen and Reich [9]. Three alternative mechanisms, which lead to the appearance of the 2D-mode, could be deduced.

**Fig. 3:**
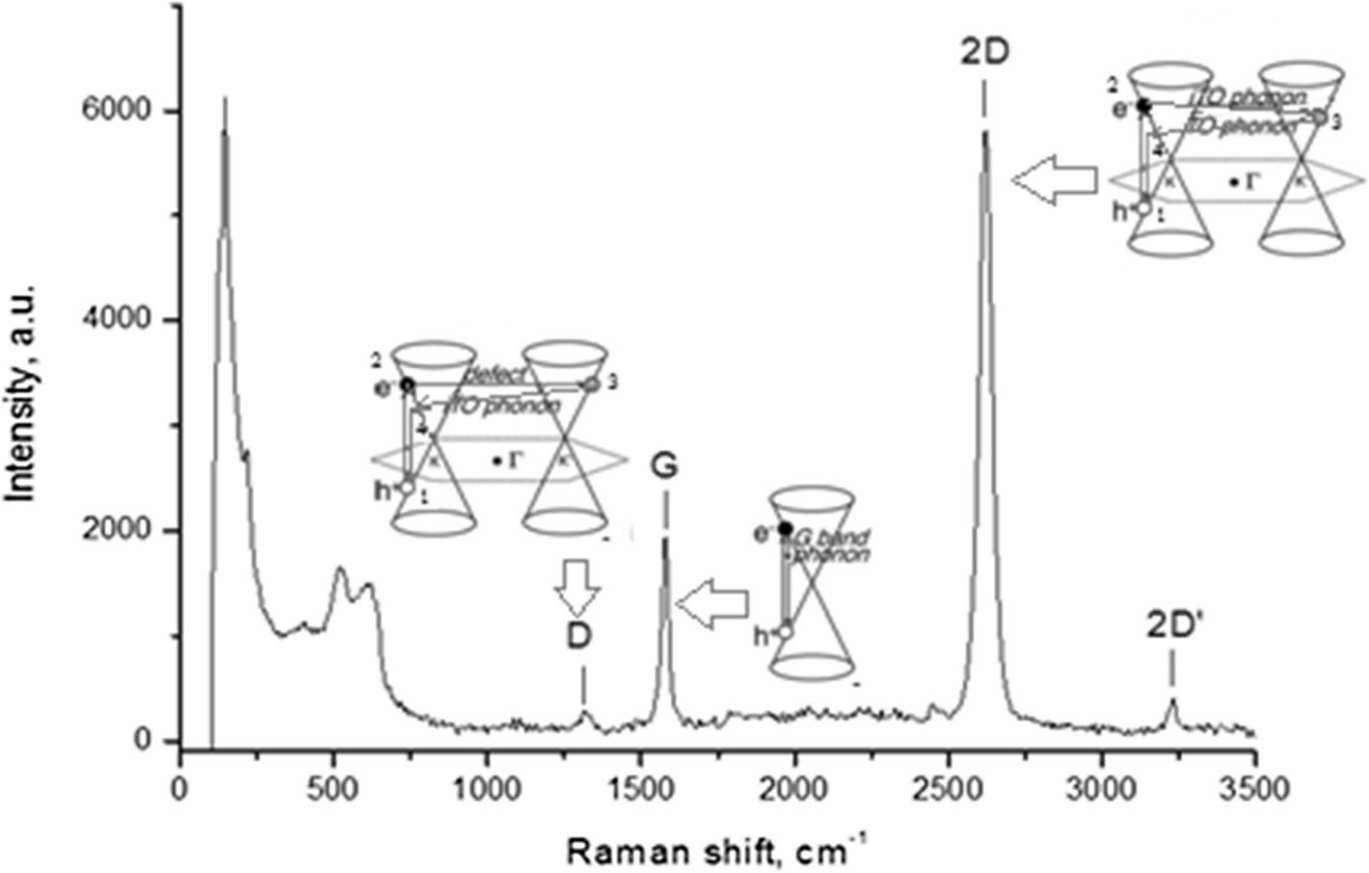
Raman spectrum of single-layer graphene on a substrate of copper foil (D-mode = 1314 cm^−1^, G-mode = 1581 cm^−1^, 2D-mode = 2621 cm^−1^, 2D′-mode = 3232 cm^−1^). Mode at 621, 521, 216, and 147 cm^−1^ corresponds to a copper foil substrate (100 % power laser 10 mW excitation at wavelength 0.63 microns)

The stages of electron-phonon processes leading to the appearance of 2D-band in the Raman spectroscopy (RS) of graphene are the following (Fig. 3):

- The laser photon excites an electron with wave vector **k**_**e**_ in the conduction zone and a hole with the wave vector **k**_**h**_ in the valence zone (the wave vectors are calculated in the *Г*-point, transition 1–2). This process could be named resonant Raman in contrast to non-resonant Raman, where the virtual states of the electron and the hole are excited.
- The electron comes to the other equivalent well and emits a phonon of i-TO branch of the dispersion curve (transition 2 → 3).
- The electron returns to the state with approximately the same wave vector (≈**k**_**e**_), but its energy decreased by the energy of the emitted phonon. As a result, another phonon is emitted (transition 3 → 4).
- The electron recombines with the hole (transition 4 → 1) and emits the Stokes Raman photon. Such process leads to a double electron-phonon resonance.
- Transitions occur simultaneously with the electron in the conductance zone (electron goes through the states 1–2–3–4–1). The hole goes through equivalent states in the valence zone.
- The electron goes through the states 1–2, 2–3 and transfers to the second well. The hole goes through the states in the valence zone simultaneously. Recombination of the electron and the hole occurs afterwards.

The transition 3 → 4 for D-band, which involves the structural defects, is a phononless. The sequence of transitions for D′- and 2D′-bands is the same, but the processes occur within one well.

In the case of G-band, a laser photon excites an electron with the wave vector **k**_**e**_ in the conductance zone and a hole with the wave vector **k**_**h**_ in the valence zone (the value is calculated in the *Г*-point). The electron emits a phonon with the wave vector **q** = 0 and recombines with a hole. All events occur in the frame of one valley.

### The Scheme of Calculation of the Wave Vector

**Fig. 4:**
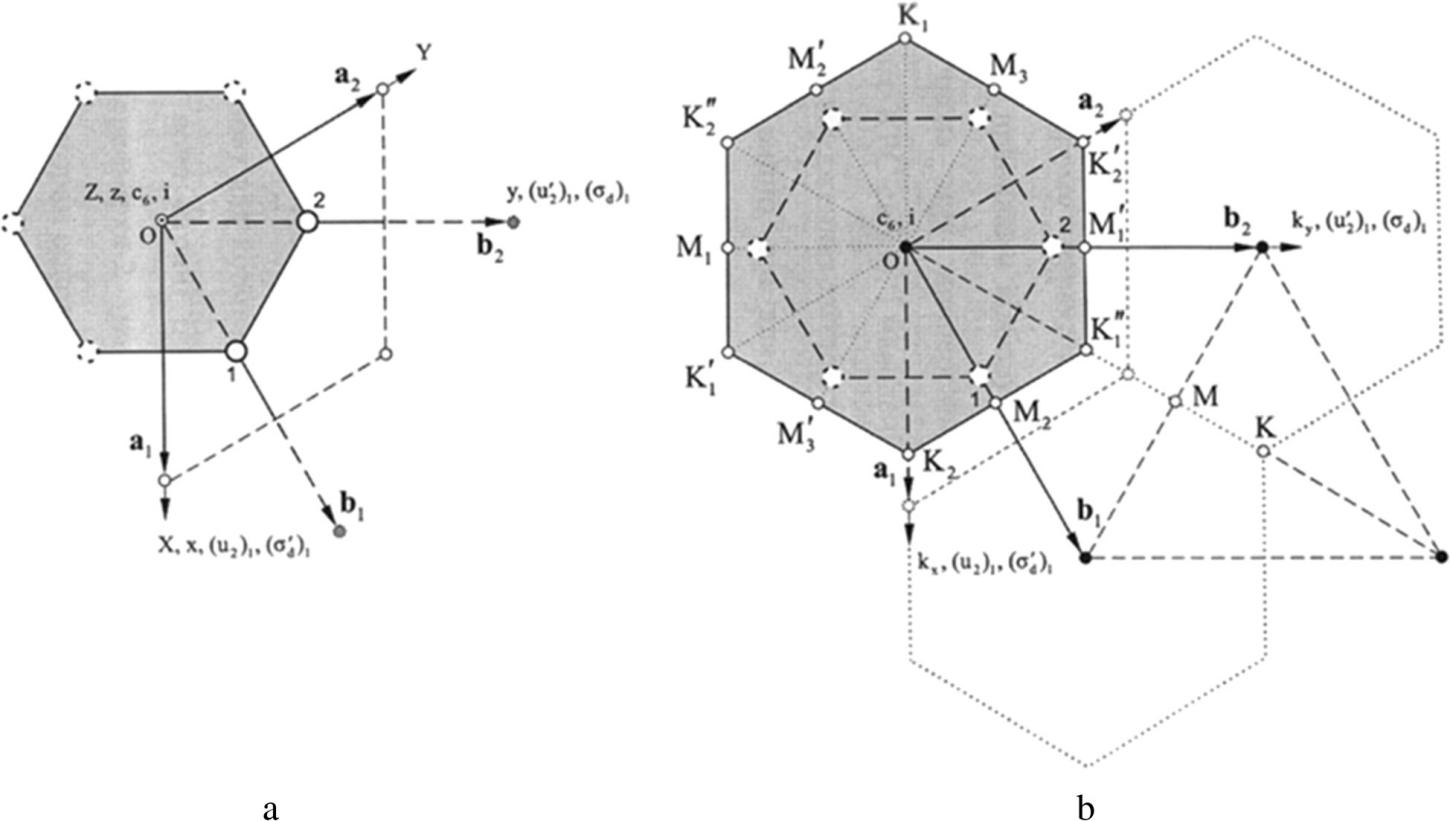
The unit cell of the Wigner-Seitz cell (**a**) and the Brillouin zone (**b**) single-layer graphene [15]

In order to calculate the wave vector of photons participating in the RS process, we computed the basic vector of reciprocal lattice (Fig. 4) as

1 $$ \left|\overrightarrow{b_1}\right|=\frac{2\pi }{ \cos \varphi \left|\overrightarrow{a_1}\right|} $$

where *a*_1_ = 24,562 Å and *φ* = 30^0^. So, *b*_1_ = 29,539 Å^−1^. The wave vector in the *M*-point was determined as *b*_1_ divided by 2 (from geometrical considerations). So, the value of wave vector at the *M*-point is *k*(M) = 14.7*10^7^ cm^−1^. The value of wave vector at the *K*-point is $k(K)=\frac{k(M)}{ \cos \varphi }=16.7*{10}^7$ cm^−1^. These values are the basis for the construction of the dispersion curve diagram of phonon states in graphene.

**Fig. 5:**
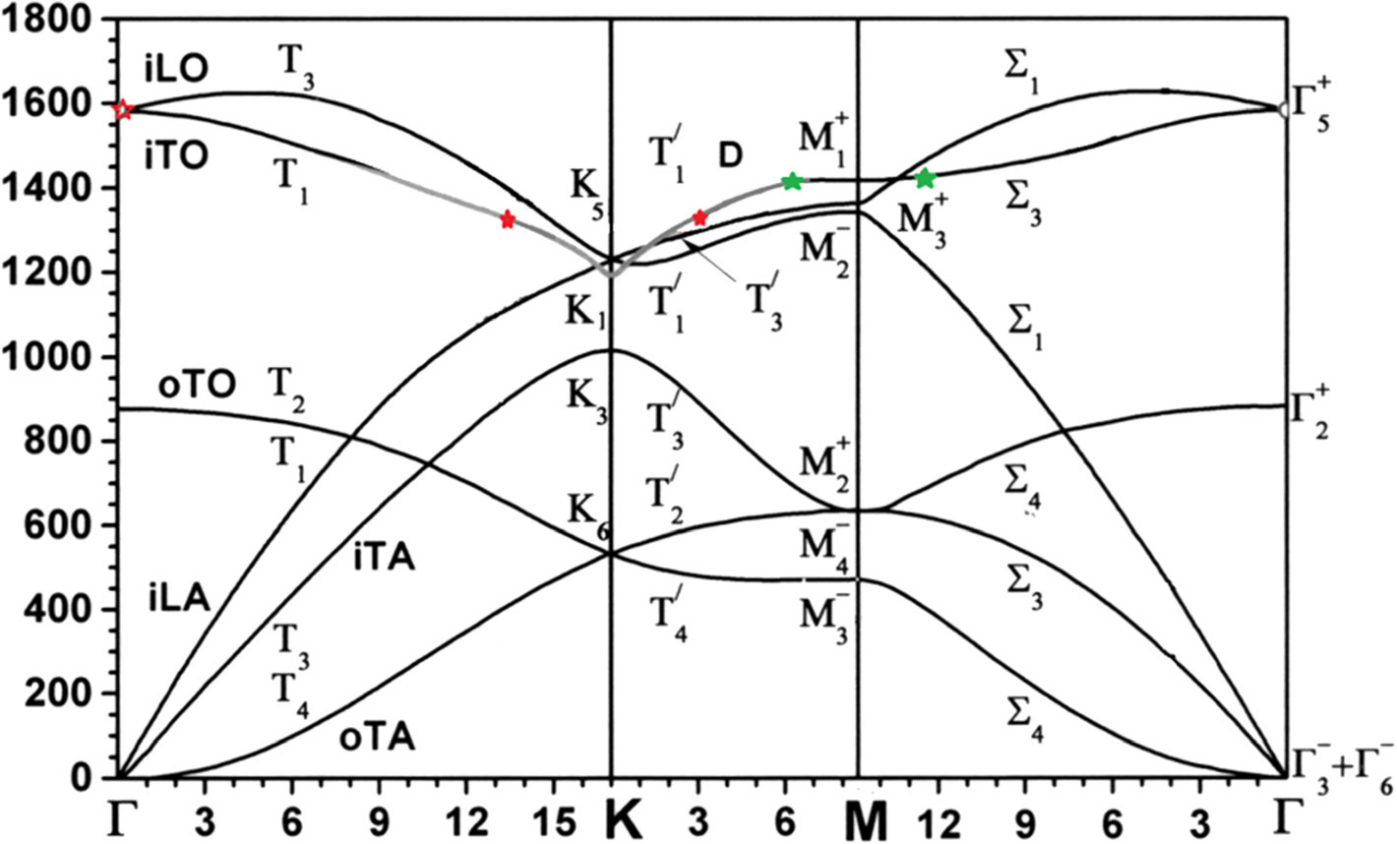
Dispersion of phonon state for one-layer graphene in ГМКГ directions

**Fig. 6:**
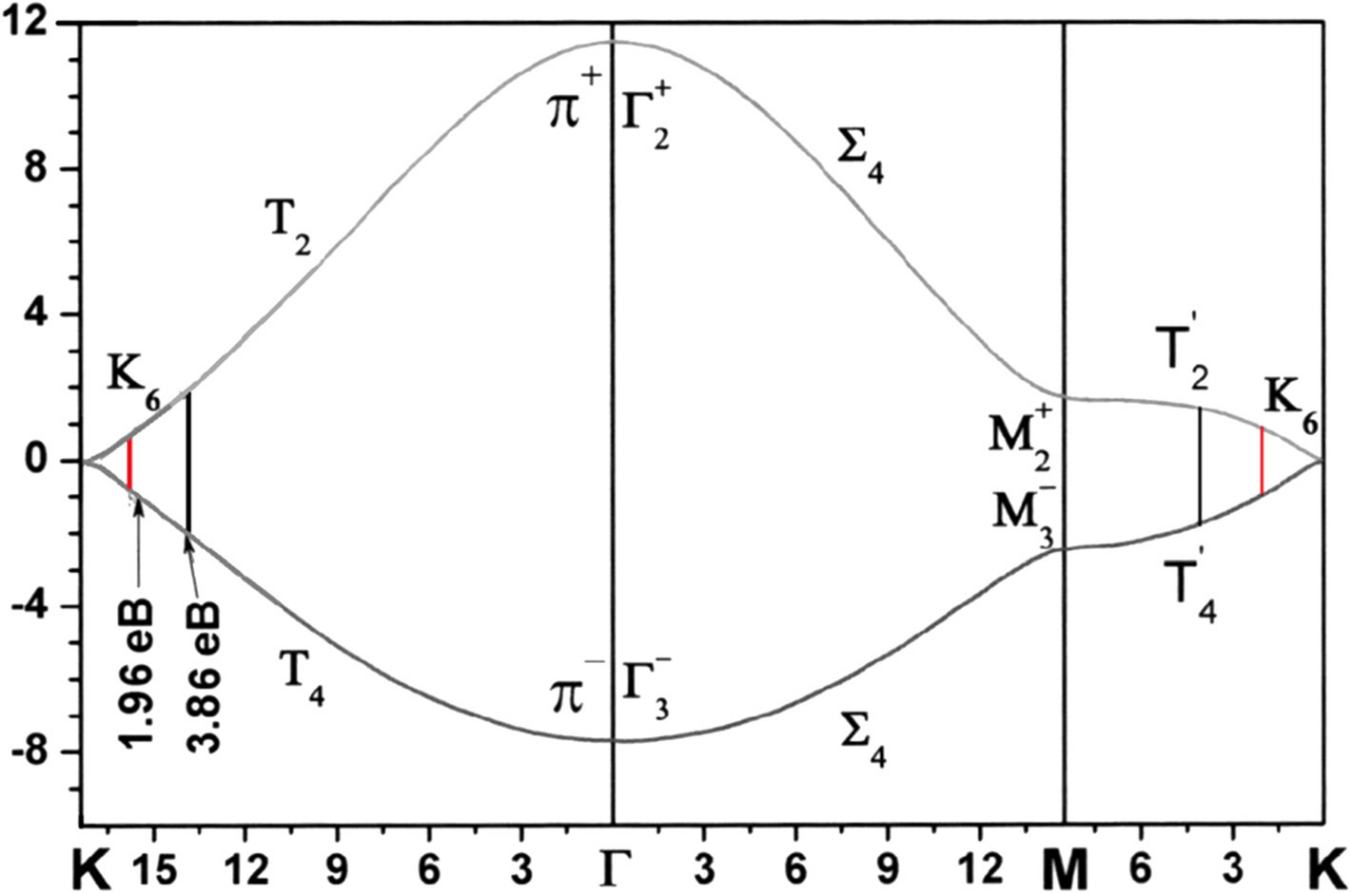
The structure of the electronic bands for single-layer graphene along the K-Г-M-K of the Brillouin zone constructed from ab-initio calculation [16] and from experiments on scattering

The values of electron wave vectors participating in Raman scattering and CARS in the vicinity of the *K*-points and *M*-points were calculated from the energy of excited photons (1.96 eV for Raman scattering and 3.86 eV for CARS) from the electron dispersion curves (Fig. 5). The theoretical curves are taken from [9]. These wave vectors were combined with experimentally determined energy of photons to reconstruct phonon dispersion curves (Fig. 6). The regions in the vicinity of the *K* point (shown in *red*) and in the vicinity of the *M* point (shown in *green*) (Fig. 6) are in good agreement with dispersion curves calculated earlier in this paper and our data (Table 1).

An enhancement factor of about 10^5^ was obtained in CARS experiment of thymine on graphene oxide [8]. Due to the fact that plasmons in graphene do not interact with light, only a special technique could excite the surface modes. The most plausible mechanism is the resonance interaction of light with the graphene-molecule complex, which has the graphite-like structure. Such complex is similar to the fragment of graphite due to π-π interaction between the molecules (Thy) and the graphene rings [10]. In this structure, the electrons have quasi-continuous number of states, so the Raman spectra could have resonant nature similar to those in pure graphene.

### Discrete Breathers

Discrete breather is a spatially localized nonlinear vibrational mode in the defect-free lattice [11, 12]. Discrete breathers may exist as long-lived oscillation modes, since they do not excite low amplitude phonons. Thus, they keep their energy because the frequency of the discrete breathers lies either above the phonon spectrum or in its gap [13]. However, the so-called embedded discrete breathers with frequencies lying in the phonon oscillation spectrum exist under specific conditions [14].

**Fig. 7:**
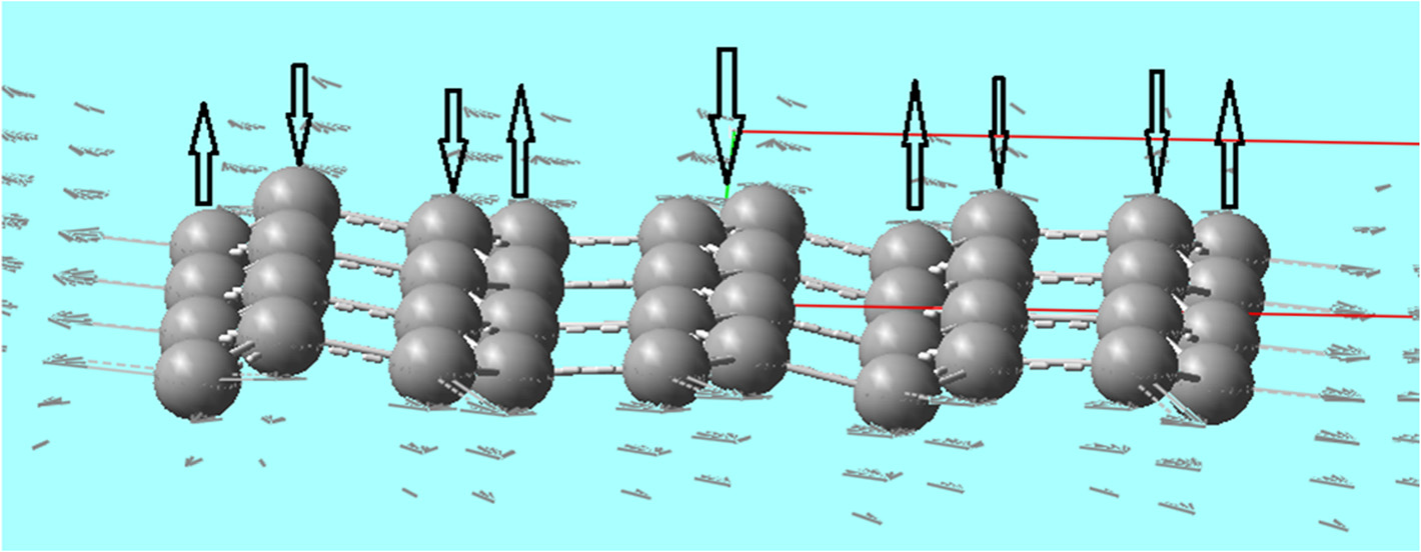
The motion of the atoms at a wavelength of supposed discrete breathers

Since the lattice dynamics in the first principle calculations is not limited to linear approximation, we can expect obtaining nonlinear excitations such as discrete breathers. In our calculations, they are observed near the frequency of 714 cm^−1^ (Fig. 7).

## Conclusions

Fundamental modes of graphene at the *Г*, *K*, and *M* points of the Brillouin zone were identified using the quantum mechanical projection operator. A correspondence between available notations for optical modes of graphene in the different points of the Brillouin zone are provided. Experimental data obtained by Raman and CARS spectroscopy are compared with calculations. The discrepancies between experimental and calculated frequencies of the vibrational spectra were analyzed. Based on these data, a resonant mechanism of RS and CARS in graphene was postulated. A possible existence of discrete breathers in graphene with the frequency of about 714 cm^−1^ was shown.
